# Factors Related to the Level of Depression and Suicidal Behavior Among Men With Diagnosed Depression, Physically Ill Men, and Healthy Men

**DOI:** 10.3389/fpsyt.2021.644097

**Published:** 2021-06-23

**Authors:** Aleksandra Kielan, Mariusz Jaworski, Anna Mosiołek, Jan Chodkiewicz, Łukasz Święcicki, Bożena Walewska-Zielecka

**Affiliations:** ^1^Department of Public Health, Faculty of Health Sciences, Medical University of Warsaw, Warsaw, Poland; ^2^Polish Suicidological Association, Warsaw, Poland; ^3^Department of Education and Research in Health Sciences, Faculty of Health Sciences, Medical University of Warsaw, Warsaw, Poland; ^4^Department of Psychiatry, Faculty of Health Sciences, Medical University of Warsaw, Pruszków, Poland; ^5^Department of Psychoprophylaxis and Addiction Psychology, Institute of Psychology, University of Lodz, Łódz, Poland; ^6^2nd Department of Psychiatry, Institute of Psychiatry and Neurology, Warsaw, Poland

**Keywords:** depressive disorders, physically ill, suicidal behavior, risk factors, protective factors

## Abstract

Depression is the most common psychiatric disorder in people who die by suicide. Awareness of risk factors for suicide in depression is important for clinicians. The study was aimed at establishing models of factors related to the level of depression and suicidal behavior among men from three different groups—in men with depressive disorder, in comparison to men with physical disorder and healthy men. A total of 598 men were included in the study. The following questionnaires were used in research model: test with sociodemographic variables, AUDIT Test, Fagerström Test, Generalized Self-Efficacy Scale (GSES), Inventory for Measuring Coping with Stress (Mini-COPE), Resilience Evaluation Questionnaire (KOP-26), Suicide Behaviors Questionnaire—Revised (SBQ-R) by Osman, and Gotland Male Depression Scale. In men with depression, the positive factors strongly related to the intensity of depression and suicidal behavior were as follows: vocational education, active coping, turning toward religion, social competence for resilience, and bachelor status. The factors negatively related to the intensity of depression and suicidal behavior in this group were as follows: unemployed status, student status, low satisfaction with the financial situation, having children, history of mental disorders in family, alcohol addiction, and seeking instrumental support. In the group of men with physical disorders, the following protection factors were identified: the medium or small city as a place of living, active coping, venting, and personal competence. The following risk factors were identified in this group: psychiatric treatment in the past. In the group of healthy men, the following protective factors were identified: the medium city as a place of living, positive reappraisal, planning abilities, and personal and social competence for resilience. In this group, the following risk factors were identified: vocational and higher education, student status, satisfaction with the financial situation, having more than one children, the occurrence of mental disorders in the family, the occurrence of alcohol abuse in the family, and use of psychoactive substances as a strategy of dealing with stress. The risk factors identified in this study should be included in the clinical assessment of depression and suicidal behavior risk in male patients. There are some protective factors identified, including productive coping and personal and social competencies, which can be developed and should be especially considered and strengthened in mental health promotion programs aimed at men.

## Introduction

Mental well-being and mental disorders result from a complex combination of events and conditions that take place in biological, individual–psychological, social–psychological, and structural domains. The interplay between the individual and the environment is crucial. The population health model encompasses the full range of risk and protective factors that determine health (at the individual; family, friend, peer; organization, community; sector/system, and society levels) (1).

Depression in men may often manifest by unspecific symptoms, which makes it difficult to establish a proper diagnosis and to estimate a real number of patients (2). Studies by Rice et al. (3) indicate six characteristic symptoms of male depression, i.e., suppression of emotions, alcohol or drug use, anger and aggression manifestation, somatic symptoms, and risky behaviors. The number of suicides in Poland (despite the noteworthy decline between 2015 and 2018) is very high, exceeding 5,000 people annually (even exceeding the number of car accident victims). Men commit suicide much more often than women (6:1) (4). Depressive disorders are connected with suicidal tendencies; in the majority of countries, suicide is one of the most frequent causes of death among depressive patients (5). More specifically, estimates indicate that ~60% of suicide victims suffered from major depressive disorder and other mood disorders (6). Masculine norms may be deleterious to the mental health of young males, placing them at greater risk of suicidal ideation (7).

The National Institute of Mental Health (8) noted that the main depressive risk factors are as follows: personal or family history of depression, major life changes, trauma, stress, certain physical illnesses, and medications. According to the World Health Organization (9), risk factors for suicide include, for instance, previous suicide attempt(s), mental health problems and disorders, problematic substance use, job loss or financial loss, trauma or abuse, and chronic pain or illness, including cancer, diabetes, and HIV/AIDS. There are also some factors that can have a protective dimension. Particularly noteworthy are individual factors related to personality (e.g., self-efficacy), environmental factors (e.g., lifestyle practice of positive coping strategies, social support in the place of residence, financial security, strong personal relationships, social competencies), and individual factors related to beliefs (e.g., religious or spiritual beliefs).

This study was aimed to determine factors strongly positively and negatively related to the intensity of depression and suicidal tendencies in the group of men diagnosed with depressive disorder, as compared to physically ill men and healthy men. In this study, we analyzed two types of predictors connected with depression and suicidal behavior—negative (risk factors) and positive (protective factors). It is the first study on depression and suicidal behavior comparing three different groups of men. Awareness of risk and protective factors for suicide in depression is especially important for clinicians (10).

## Materials and Methods

### Material

The studies were conducted in three groups: Group I (clinical group), group II (first control group—CG1), and group III (second control group—CG2). The study inclusion criteria for the clinical group (group I) were as follows: (1) age at least 18 years, (2) male gender, (3) diagnosis of depressive disorders (F31—in the depressive phase, F32, and F33), (4) undergoing pharmacological and psychotherapeutic treatment in the 2nd Clinic Psychiatric Institute of Psychiatry and Neurology in Warsaw or Mazowieckie Specialist Health Center prof. Jan Mazurkiewicz, and (5) informed consent to participate in the study.

The study inclusion criteria for the first control group (CG1) were as follows: (1) age at least 18 years, (2) male gender, (3) no current pharmacological and psychotherapeutic treatment at any psychiatric hospital, (4) informed consent to participate in study, and (5) currently undergoing treatment of any physical illness in four randomly selected hospitals in Warsaw at randomly selected departments. Patients from randomly selected hospitals in Warsaw were selected for the first control group to collect responses from men with no history of depressive disorder. The randomness of hospitals and departments was used to reduce the error resulting from the health condition of the male respondents from the control group. Patients qualified for the first control group stayed in the following hospitals:

1. Samodzielny Publiczny Centralny Szpital Kliniczny (Independent Public Central Clinical Hospital), Department of Internal Diseases, Pneumonology and Allergology, (*N* = 45).
2. Wojskowy Instytut Medyczny Centralny Szpital Kliniczny MON (Military Institute of Medicine Central Clinical Hospital of the Ministry of National Defense), Department of Internal Medicine, Nephrology and Dialysis, (*N* = 40).
3. Miedzyleski Szpital Specjalistyczny (Miedzyleski Specialist Hospital), Department of Adult Dermatology, (*N* = 48).
4. Mazowiecki Szpital Bródnowski (Mazowiecki Bródnowski Hospital), Traumatic and Orthopedic Surgery and Rehabilitation Department Complex, (*N* = 50).

The study inclusion criteria for the second control group (CG2) were as follows: (1) age at least 18 years, (2) male gender, (3) no current pharmacological and psychotherapeutic treatment at any psychiatric hospital, (4) informed consent to participate in study, and (5) feeling physically and mentally healthy. The snowball method was used to include the participants in this group.

A total of 598 questionnaires were collected: 197 from the clinical group (CG, group I), 198 from CG1, and 203 from CG2. For CG1, Gotland Male Depression Scale revealed that 1 person showed depression syndromes and 14 men met the criteria of possible depression (for CG2: 1 person with depression and 5 with possible depression). Therefore, those 15 persons (7.6%) were excluded from CG1 (6 persons, 3.0% from CG2) and further analysis included only men with no signals of depression in control groups (*n* = 183 for CG1 and *n* = 197 for CG2).

Sociodemographic characteristics of study groups are presented in Table 1. Men in CG1 were significantly older (47.97 ± 17.40 years) than in CG (44.14 ± 14.27 years) and CG2 (42.99 ± 14.48 years) with weak effect size, η^2^ =.02; *p* = 0.005. Education, place of living, professional activity, and marital status were also significantly different between groups with weak scale effect (*V* from 0.21 to 0.30; *p* < 0.001 for each characteristic). Patients from villages and small cities were found more frequent in CG than in other groups (in total 36 vs. 15% in CG1 and 5% in CG2). Primary and secondary education was more frequently noted in CG, while vocational and higher education was noted in control groups. Responders from control groups were more often professionally active than CG patients (52% in CG vs. 64% in CG1 and 80% in CG2) and were more frequently married (34% in CG vs. 59% in CG1 and 56% in CG2). Patients from the clinical group (CG) were more often unemployed or bachelors than in control groups. The number of children was significantly different among groups with small scale effect (52% in CG vs. 68% in CG1 and 60% in CG2; *V* = 0.13; *p* = 0.009).

**Table 1:** Sociodemographic characteristic of study groups.

**Characteristic**	**Clinical group**	**Control group 1 (somatic disease—CG1)**	**Control group 2 (healthy—CG2)**	**Effect size**	***p***
N	197	183	197		
Age, years	44.14 ± 14.27	47.97 ± 17.40	42.99 ± 14.48	0.02	0.005
Place of living					
Large city (>200k habitants)	84 (42.6)	110 (60.1)	155 (78.7)	0.26	<0.001
Medium city (50k−200k habitants)	41 (20.8)	45 (24.6)	33 (16.8)		
Small city (<50k habitants)	41 (20.8)	13 (7.1)	6 (3.0)		
Village	31 (15.7)	15 (8.2)	3 (1.5)		
Education					
Primary	20 (10.2)	3 (1.6)	12 (6.1)	0.23	<0.001
Secondary	87 (44.2)	41 (22.4)	39 (19.8)		
Vocational	38 (19.3)	65 (35.5)	44 (22.3)		
Higher	52 (26.4)	74 (40.4)	102 (51.8)		
Work status					
Student	8 (4.1)	7 (3.8)	17 (8.6)	0.30	<0.001
Unemployed	34 (17.3)	2 (1.1)	1 (0.5)		
Working	101 (51.5)	117 (63.9)	157 (79.7)		
Retired/pensioner	43 (21.9)	56 (30.6)	21 (10.7)		
Dependence on another family member	10 (5.1)	1 (0.5)	1 (0.5)		
Marital status					
Married	66 (33.5)	107 (58.5)	110 (55.8)	0.21	<0.001
Separated/divorced	33 (16.8)	11 (6.0)	17 (8.6)		
Widower	8 (4.1)	10 (5.5)	6 (3.0)		
Bachelor	66 (33.5)	29 (1.8)	29 (14.7)		
Informal relationship	23 (11.7)	26 (14.2)	35 (17.8)		
Having kids	102 (52.0)	123 (67.6)	119 (60.4)	0.13	0.009
Number of kids	2.00 (1.00;2.00)	2.00 (1.25;2.00)	1.00 (1.00;1.00)	0.004	0.320

### Instruments

The survey included standardized screening questionnaires and socio-demographic variables with few additional questions (Have you ever had thoughts of suicide? Have you ever tried to take your own life? Have you had/are there any mental disorders in your family? Have you ever been treated psychiatrically? The last question was detailed with the number of treatment episodes, a diagnosis that has been identified in the course of treatment, and the extent of treatment support received from a family ranging from very high, high, moderate, low to none).

The following standardized questionnaires were used in this study:

1. AUDIT test—the Alcohol Use Disorders Identification Test

A test commissioned by the World Health Organization, consisting of 10 questions arranged in three parts: the first part evaluates risky drinking (frequency of drinking, the average amount of alcohol drunk, frequency of excessive drinking), the second part evaluates symptoms of addiction (loss of control over drinking, drinking as an increasingly important issue in life, the need to drink in the morning), and the third part contains questions on harmful drinking (feeling guilty after alcohol drinking, memory lapses caused by drinking, physical injuries caused by alcohol drinking, the concern of others about your drinking) (11). The Polish version of AUDIT was used (Cronbach's α = 0.78 in the original study and 0.903 in our study) (12).

2. Fagerström Test

The Fagerström Nicotine Addiction Test consists of six questions, which can be scored from 0 to 10. A score from 0 to 2 means a very low level of addiction; score 3–4, low; score 5, moderate; score, 6–7, high; score 8–10, very high level of addiction (13). The Polish version of the Fagerstrom Test was used—Cronbach's α = 0.76 in the original study and 0.65 in our study (14).

3. GSES—Generalized Self-Efficacy Scale

The scale by Schwarzer, Jerusalem, and Juczyński in the Polish adaptation by Juczyński (15). The scale consists of 10 statements, which determined the level of self-efficacy of the examined person—Cronbach's α = 0.85 in the original study and 0.908 in our study.

4. MINI-COPE Questionnaire (Brief COPE Inventory)

Coping strategies were measured by the MINI-COPE Questionnaire (Brief COPE Inventory) (16) in the Polish adaptation by Juczyński and Ogińska-Bulik (15). There are 28 statements integrated into 14 coping strategies (i.e., two statements per strategy): active coping, planning, positive reframing, acceptance, sense of humor, religious solace, use of emotional support, use of instrumental support, self-distraction, denial, venting, substance abuse, behavioral disengagement, and self-blame. The respondent selected one out of four possible answers ranging in scores from “I have almost never been doing this” (0 points) to “I have almost always been doing this” (3 points). Each of the coping strategies was assessed separately, and the higher the score, the more often a particular strategy was adopted. Depending on subscale, Cronbach's α = 0.45–0.82 in the original study and 0.484–0.912 in our study.

5. Resilience Evaluation Questionnaire (KOP-26)

This questionnaire was created by Gasior, Chodkiewicz, and Cechowski (17). The questionnaire consists of 26 items. The assessment of the extent to which the respondent agrees with a given statement is made on a five-point Likert scale [from 1 (I completely disagree) to 5 (I completely agree)]. Four variables are assessed based on the questionnaire: personal competencies, family competencies, social competencies, and general resilience (assumed by all three types of competencies). The Polish version of KOP-26 was used. Cronbach's α = 0.90 for total scale, 0.78–0.90 for subscales in the original study and 0.943 for total scale, 0.847–0.925 for subscales in our study.

6. Suicide Behaviors Questionnaire-Revised (SBQ-R) by Osman

The scale created by Osman et al. (18) in the Polish adaptation by Chodkiewicz and Gruszczyńska (19). The questionnaire is a self-descriptive measure of suicidal tendencies (suicidal behaviors, including ideation and attempts) composed of four questions. Cronbach's α = 0.83 in the Polish adaptation study and 0.866 in our study.

7. Gotland Male Depression Scale

The scale was created by Rutz (20) in the Polish adaptation by Chodkiewicz (21). A scale consists of 13 statements describing the depressive symptoms of the people examined a month before. Each of the statements is scored on the four-point Likert scale: from 0 (“completely untrue”) to 3 (“completely true”). The overall result is in the range from 0 to 39 points. The authors adopted the following interpretation of the results on the full scale: 0–12 points, no signs of depression; 13–26, possible depression, appropriate treatment should be considered; 27–39, depression, most likely treatment is necessary (including pharmacological). Cronbach's α = 0.85 in the Polish adaptation study and 0.948 in our study).

### Procedure

The study was carried out in 2018–2020 using the paper-pencil method. The researchers obtained approval from the management of all hospitals to conduct the study on its premises. The researcher, after making an appointment with the head of a ward participating in the study, was introduced to the ward staff and later was introduced to patients selected by the head of the ward, staying in the ward (the patients' state of health had to allow obtaining informed consent to participate in the study). The researcher introduced himself and discussed the study participation procedure. Before providing the patient with a questionnaire to complete, informed consent to participate in the study was obtained from each patient. It took the patients about 20–25 min to complete the questionnaire. The survey was voluntary and anonymous.

The collected and digitized data was stored by the researcher under the guidelines in force at the Medical University of Warsaw.

### Ethical Declarations

The study was approved by the Bioethics Committee of Warsaw Medical University No. KE-0254/335/2015.

### Statistical Analysis

For ease of reading, study groups were described as CG (clinical group), CG1 (somatic disease, mentally healthy), and CG2 (physically and mentally healthy).

Multivariate regression was used for each of the groups (TG, CG1, CG2) separately to identify factors impacting the level of depression. At first, we performed univariate regression models with all predictor variables and with GMDS and SBQ-R as outcome variables. Separate models were created to model the level of depression (based on GMDS score) and risk level of suicidal behaviors (based on SBQ-R score). Based on univariate models, into the final multivariate models, only these predictor variables were included, which were statistically significant in univariate regression models. A stepwise approach was used. Additionally, we verified the level of multicollinearity between predictor variables using variance inflation factors (VIFs) and values above 10 indicating high multicollinearity (22). Finally, R-squared was calculated to assess the explanatory power of each model.

Apart from the multivariate model in each group, additional models using logistic regression analysis were created for depression level to identify the group of factors predicting the presence of depression. One model included predictor variable understood as 1 = clinical group patients with depression/potential depression; 0 = control group 1 patients (somatic disease patients without depression). The second model included predictor variable understood as 1 = clinical group patients with depression/potential depression; 0 = control group 2 patients (healthy patients without depression). At first, we performed univariate logistic regression models with all predictor variables, based on which we included into the final multivariate models only these predictor variables that expressed *p* < 0.25 in univariate regression models, as per Hosmer and Leweshow recommendations (23). A stepwise approach with AIC criterion was used. As logistic regression was used, model coefficients were presented as log-odds (i.e., when the predictor increases by one unit, the outcome increases by log-odds). For ease of interpretation, log odds were exponentiated into odds ratios (OR) such that when the predictor increases one unit, the expected change in the outcome is described in terms of % odds. Models' evaluation was based on the R2 Nagelkerky coefficient and Hosmer and Lemeshow goodness of fit (GOF) test.

All tests were two-tailed, and results were regarded as statistically significant at the level of *p* <.05. Analysis was conducted in statistical software R (version 3.5.1).

## Results

Multivariate regression analysis confirmed that in the clinical group, the following variables were significant predictors of depression (GMDS level): education vocational vs. primary, β = −3.01; *CI*_95_ (−5.15 to −0.86]; *p* = 0.006, unemployed vs. working work status, β = 2.51; *CI*_95_ (1.02–3.99); *p* = 0.001, student vs. working work status, β = 3.42; *CI*_95_ (0.62–6.23); *p* = 0.017, satisfaction from financial situation, β = 0.60; *CI*_95_ (0.09–1.10); *p* = 0.020, having kids, β = 1.64; *CI*_95_ (0.40–2.88); *p* = 0.010, presence of mental disorders in family, β = 2.49; *CI*_95_ (1.30–3.68); *p* < 0.001, alcohol addiction AUDIT, β = 0.14; *CI*_95_ (0.08–0.20); *p* < 0.001, active coping strategy of dealing with stress, β = −1.31; *CI*_95_ (−2.22 to −0.40); *p* = 0.005, turning to religion strategy of dealing with stress, β = −0.94; *CI*_95_ (−1.52 to −0.37); *p* = 0.001, seeking instrumental support strategy of dealing with stress, β = 1.18; *CI*_95_ (0.37–2.00); *p* = 0.005, and social competence for resilience, β = −0.36; *CI*_95_ (−0.49 to −0.22); *p* < 0.001. The model explained 47% of variation in depression variable.

Similar model for CG1 group resulted in the following range of variables predicting significant depression level: medium vs. large city place of living, β = −0.58; *CI*_95_ (−1.14 to −0.01); *p* = 0.047, small city vs. large city place of living, β = −0.95; *CI*_95_ (−1.87 to −0.03); *p* = 0.043, psychiatric treatment in the past, β = 3.51; *CI*_95_ (1.27–5.76); *p* = 0.002, and active coping strategy of dealing with stress, β = −0.74; *CI*_95_ (−1.27 to −0.20); *p* = 0.007. The model explained only 13% of variation in depression variable.

Depression level in CG2 group was significantly modeled by the following: medium vs. large city place of living, β = −0.83; *CI*_95_ (−1.40 to −0.25); *p* = 0.005, education: vocational vs. primary, β = 1.67; *CI*_95_ (0.69–2.64); *p* = 0.001, education: higher vs. primary, β = 1.38; *CI*_95_ (0.40–2.35); *p* =.006, professional activity: student vs. professionally active was concerned, β = 2.00; *CI*_95_ (0.90–3.10); *p* < 0.001, satisfaction with financial situation, β = 0.79; *CI*_95_ (0.46–1.12); *p* < 0.001, number of children, β = 0.23; *CI*_95_ (0.01–0.45); *p* = 0.042, occurrence of mental disorders in family, β = 1.39; *CI*_95_ (0.68–2.10); *p* < 0.001, occurrence of alcohol abuse in family, β = 0.84; *CI*_95_ (0.33–1.35); *p* = 0.001, positive reappraisal strategy of dealing with stress, β = −0.86; *CI*_95_ (−1.29 to −0.44); *p* = 0.001, and use of psychoactive substances as a strategy of dealing with stress, β = 0.69; *CI*_95_ (0.25–1.04); *p* < 0.001. The model explained 43% of variation in depression variable (Table 2).

**Table 2:** Multivariate regression analysis for depression severity at men.

**Multivariate regression model for depression severity**	**Clinical group**				**Control group 1 (somatic disease)**				**Control group 2 (healthy)**			
	**β**	**95% CI for β**	**B**	***P***	**β**	**95% CI for β**	**B**	***p***	**β**	**95% CI for β**	**B**	***p***
Place of living, Large cities = baseline medium city					−0.58	−1.14 to −0.01	−0.35	0.047	−0.83	−1.40 to −0.25	−0.47	0.005
Small city					−0.95	−1.87 to −0.03	−0.57	0.043				
Education, Primary = baseline vocational	−3.01	−5.15 to −0.86	−0.66	0.006					1.67	0.69–2.64	0.85	0.001
Higher									1.38	0.40–2.35	0.78	0.006
Work status, Working (professionally active) = baseline	2.51	1.02–3.99	0.55	0.001								
Unemployed												
Student	3.42	0.62–6.23	0.76	0.017					2.00	0.90–3.10	0.94	<0.001
Financial situation satisfaction	0.60	0.09–1.10	0.16	0.020					0.79	0.46–1.12	0.41	<0.001
Having children	1.64	0.40–2.88	0.18	0.010								
Number of children									0.23	0.01–0.45	0.14	0.042
Psychiatric treatment in the past					3.51	1.27–5.76	0.23	0.002				
Mental disorders in family (1 = yes)	2.49	1.30–3.68	0.26	<0.001					1.39	0.68–2.10	0.25	<0.001
Alcohol abuse in family (1 = yes)									0.84	0.33–1.35	0.20	0.001
Alcohol addiction (by AUDIT)	0.14	0.08–0.20	0.29	<0.001								
Mini-COPE Active coping	−1.31	−2.22 to −0.40	−0.19	0.005	−0.74	−1.27 to −0.20	−0.20	0.007				
Positive reappraisal									−0.86	−1.29 to −0.44	−0.32	0.001
Turning to religion	−0.94	−1.52 to −0.37	−0.21	0.001								
Seeking instrumental support	1.18	0.37–2.00	0.20	0.005								
Use of psychoactive substances									0.69	0.25–1.04	0.27	<0.001
Social competence	−0.36	−0.49 to −0.22	−0.42	<0.001								
Constant	8.54	4.53–12.56	0.21	<0.001	6.02	4.80–7.24	0.14	<0.001	−4.93	−8.19 to −1.67	−0.66	0.011
R2	0.47				0.13				0.43			
R2 adj.	0.42				0.11				0.36			
VIF range	From 1.23 to 1.90				From 1.02 to 1.03				From 1.11 to 4.65			

*β, beta coefficient (non-standardized); 95% CI for β, 95% confidence interval for beta coefficient; B, beta coefficient (standardized); VIF, variance inflation factors*.

Multivariate regression analysis confirmed that in the clinical group (CG) the following variables were identified as significant predictors of suicidal behaviors: professionally status: unemployed vs. professionally active, β = 3.15; *CI*_95_ (1.73–4.58); *p* < 0.001, working status student vs. professionally active, β = 3.14; *CI*_95_ (0.24–6.05); *p* = 0.034, marital status: bachelor vs. married, β = −1.80; *CI*_95_ (−3.22 to −0.38); *p* = 0.013, occurrence of mental disorders in family, β = 2.03; *CI*_95_ (0.79–3.27); *p* = 0.001, active coping strategy of dealing with stress, β = −1.09; *CI*_95_ (−2.02 to −0.17); *p* = 0.021, turning toward religion as a strategy of dealing with stress, β = −0.79; *CI*_95_ (−1.39 to −0.20); *p* = 0.009, seeking instrumental support strategy of dealing with stress, β = 0.97; *CI*_95_ (0.17–1.78]; *p* = 0.018, and social competence for resilience, β = −0.34; *CI*_95_ (−0.49 to −0.20); *p* < 0.001. The model explained 48% of variation in suicidal behaviors variable.

In the CG1 group, a similar model resulted in the following range of variables predicting significant suicidal behaviors risk level: place of living: medium vs. large city, β = −0.60; *CI*_95_ (−1.17 to −0.03); *p* = 0.038, psychiatric treatment in the past, β = 4.05; *CI*_95_ (1.81–6.29); *p* = 0.001, active coping strategy of dealing with stress, β = −0.75; *CI*_95_ (−1.32 to −0.19); *p* = 0.009, venting of emotions as a strategy of dealing with stress, β = −0.62; *CI*_95_ (−1.12 to −0.12); *p* = 0.015, and personal competence of resilience, β = −0.09; *CI*_95_ (−0.18 to −0.01); *p* = 0.038. The model explained 23% of variation in suicidal behavior variable.

In the CG2 group, the suicidal behavior risk level was significantly modeled by the following: place of living: medium vs. large city, β = −0.97; *CI*_95_ (−1.52 to −0.41); *p* = 0.001, satisfaction with financial situation, β = 0.68; *CI*_95_ (0.38–0.97); *p* < 0.001, occurrence of alcohol abuse in family, β = 0.82; *CI*_95_ (0.30–1.33); *p* = 0.002, planning strategy of dealing with stress, β = −0.66; *CI*_95_ (−1.21 to −0.12); *p* = 0.017, use of psychoactive substances as a strategy of dealing with stress, β = 0.67; *CI*_95_ (0.31–1.02); *p* < 0.001, total resilience, β = 0.11; *CI*_95_ (0.07–0.16); *p* < 0.001, personal competence of resilience, β = −0.22; *CI*_95_ (−0.33 to −0.12); *p* < 0.001, and social competence of resilience, β = −0.12; *CI*_95_ (−0.18 to −0.05); *p* = 0.001. The model explained 38% of variation in suicidal behaviors variable (Table 3).

**Table 3:** Multivariate regression analysis for suicidal behaviors at men.

**Multivariate regression model for SBQ-R**	**Clinical group**				**Control group 1 (somatic disease)**				**Control group 2 (healthy)**			
	**β**	**95% CI for β**	**B**	***p***	**β**	**95% CI for β**	**B**	***p***	**β**	**95% CI for β**	**B**	***p***
Place of living, Large cities = baseline Medium city					−0.60	−1.17 to −0.03	−0.36	0.038	−0.97	−1.52 to −0.41	−0.55	0.001
Work status, Working (professionally active) = baseline	3.15	1.73–4.58	0.70	<0.001								
Unemployed												
Student	3.14	0.24–6.05	0.70	0.034								
Financial situation satisfaction									0.68	0.38–0.97	0.35	<0.001
Marital status, Married = baseline Bachelor	−1.80	−3.22 to −0.38	−0.40	0.013								
Psychiatric treatment in the past					4.05	1.81–6.29	0.26	0.001				
Mental disorders in family (1 = yes)	2.03	0.79–3.27	0.45	0.001								
Alcohol abuse in family (1 = yes)									0.82	0.30–1.33	0.47	0.002
Mini-COPE Active coping	−1.09	−2.02 to −0.17	−0.16	0.021	−0.75	−1.32 to −0.19	−0.21	0.009				
Planning									−0.66	−1.21 to −0.12	−0.17	0.017
Religion	−0.79	−1.39 to −0.20	−0.17	0.009								
Use of instrumental support	0.97	0.17–1.78	0.16	0.018								
Venting					−0.62	−1.12 to −0.12	−0.21	0.015				
Substance use									0.67	0.31–1.02	0.26	<0.001
Resilience (KOP-26) Personal competence					−0.09	−0.18 to −0.01	−0.18	0.038	−0.22	−0.33 to −0.12	−0.58	<0.001
Social competence	−0.34	−0.49 to −0.20	−0.40	<0.001					−0.12	−0.18 to −0.05	−0.29	0.001
Constant	9.44	5.51–13.38		<0.001	8.23	5.22–11.23	0.13	<0.001	−0.09	−3.14–2.97	0.004	0.955
R2	0.48				0.23				0.38			
R2 adj.	0.40				0.17				0.34			
VIF range	From 1.30 to 2.12				From 1.10 to 1.51				From 1.11 to 7.67			

*β, beta coefficient (non-standardized); 95% CI for β, 95% confidence interval for beta coefficient; B, beta coefficient (standardized); VIF, variance inflation factors*.

Additional logistic regression models were created for depression evaluation. Factors significantly increasing the risk of depression in the clinical group (CG) vs. healthy patients were as follows: living in a small city vs. large city, or living in a village vs. large city, the occurrence of mental disorders or alcohol abuse in the family, nicotine addiction as evaluated by Fagerström questionnaire, alcohol addiction as evaluated by AUDIT questionnaire, and turning toward religion as a strategy of dealing with stress. At the same time, working vs. non-working and GSES significantly decreased the risk of depression in the same group. Another model predicting the risk of depression in the clinical group (CG) vs. mentally healthy or men with somatic illness resulted in the following significant predictors of depression: occurrence of alcohol abuse in the family, nicotine addiction (by Fagerström questionnaire), alcohol addiction (by AUDIT), and self-blame strategy of dealing with stress (all of them increased the risk of depression), as well as GSES, the strategy of positive reappraisal strategy of dealing with stress, use of psychoactive substances strategy of dealing with stress, and social competence decreased the risk of depression (Table 4). Both logistic regression models had a good fit to the data (GOF test for model 1 with *p* = 0.087 and model 2 with *p* = 0.389). Nagelkerke R2 level also confirms a good quality of both models (R2 for model 1 was 0.75, while for model 2 R2 = 0.78).

**Table 4:** Multivariate logistic regression analysis for depression severity at men.

**Characteristic**	**Model 1 (CG vs. CG2)**			**Model 2 (CG vs. CG1)**		
	***OR***	**95% *CI* for *OR***	***p***	***OR***	**95% *CI* for *OR***	***p***
Small city	11.47	2.34–67.68	0.004			
Village	34.36	6.53–220.66	<0.001			
Work status, Not-working = baseline Working	0.27	0.11–0.64	0.004			
Mental disorders in family (1 = yes)	6.32	2.35–17.73	0.003			
Alcohol abuse in family (1 = yes)	3.28	1.34–8.27	0.010	6.44	2.43–18.50	<0.001
Nicotine addiction (Fagerström)	1.41	1.21–1.67	<0.001	1.61	1.37–1.94	<0.001
Alcohol addiction (AUDIT)	1.10	1.04–1.17	0.001	1.29	1.17–1.45	<0.001
Self-efficacy (GSES)	0.81	0.74–0.87	<0.001	0.81	0.70–0.92	0.001
Mini-COPE						
Positive reappraisal				0.16	0.06–0.40	<0.001
Turning to religion	2.50	1.64–3.92	<0.001			
Use of psychoactive substances				0.42	0.23–0.75	0.005
Self-blame				3.22	1.65–6.65	0.001
Social competence				0.87	0.77–0.98	0.027
Constant	40.01	3.09–619.76	0.06	4,957	113–372,284	<0.001

*OR, odds ratio; 95% CI for OR, 95% confidence interval for odds ratio*.

## Discussion

In-house study shows that there are some common depression risk factors for the group of men: student status, low satisfaction with the financial situation, having children/more children, and the occurrence of mental disorders in the family. There are some risk factors of depression that are characteristic only for men with this type of mental disorder: unemployment, alcohol abuse/addiction, and seeking instrumental support in coping with stress. Some of these risk factors coincide with risk factors for suicidal behavior for men with diagnosed depression, which include unemployment, student status, the occurrence of mental disorders in the family, and seeking instrumental support.

The present study noted the key role of socioeconomic factors in the development of depression. This is consistent with the results of other studies. Socioeconomic status has been found to play an important role in depression (24). A meta-analysis of population-based surveys confirmed the association of increasing intensity of depression symptoms with decreasing social position (especially for income level) (25). According to a study conducted among male truck drivers by Da Silva-Júnior et al. (26) wage-earning (OR = 2.84; *P* = 0.01) is one of the main risk factors of depression. In our study, men were assessing their satisfaction with the financial situation, which can reflect their material aspects of socioeconomic status—low satisfaction with the financial situation turned out to be a risk factor of depression among men with diagnosed depression and in the healthy group. It should be noted that the increased risk of depression in men with low income may be directly related to the workload and working conditions. Other studies emphasize that working conditions are a strong determinant of depressive disorders in men. For example, work overload under unfavorable conditions can translate into job satisfaction as well as financial satisfaction (27).

Unemployment is another socioeconomic variable that is associated with the subjective assessment of financial satisfaction. Our study shows that unemployment is one of the risk factors related to the level of depressions and suicidal behavior for men with diagnosed depressive disorder. Suzuki et al. (28) proofed that the risk of depressive tendencies is significantly higher in men who were not working (OR, 3.57; 95% CI, 1.31–9.72). Evidence suggests that the risk of depression increases steadily for 6 months after the individual becomes unemployed, then reaches a plateau and is reversed almost immediately on finding work (29). Unemployment is associated also with greater depressive symptoms, and this relationship is only observed in men and not in women (30). During unemployment, there is a critical time between 3 months and a year during which people may be the most at risk of mental disorder. This time is thought to coincide with the growing sense of hopelessness that may accompany the perceived transition from short- to long-term unemployment. The long-term unemployed have a greater risk of suicide and attempted suicide compared to those unemployed for the short term (31). Male suicide mortality increased linearly with the length of unemployment. Men may have been more likely to derive social status and prestige from their work. The male role as the primary breadwinner, either perceived or real, appears to have given unemployment a greater negative effect on men than on women (32). Individuals in poor health are at increased risk of unemployment and also suicide. The higher relative risk of suicide among the unemployed seems to be, in part, a consequence of the exclusion of less healthy individuals from the labor market (33).

The present study also shows the important role of the level of education. Other researchers emphasized that level of education is another component of socioeconomic status (34). Our results show that student status increases the level of depression and suicidal behavior among healthy men and men who have depression disorders. Scientific reports indicate a frequent occurrence of depression in students. According to a systematic review of studies of depression prevalence in university students, there is wide variation in the proportion of students identified as depressed, from relatively low rates around 10% to high rates of between 40 and 84% with a weighted mean prevalence of 30.6%. It was also estimated that the prevalence of depression among male students is about 24.9% (35). A study by Akhtar-Danesh and Landeen (36) showed that the lowest rates of depression based on the level of education were seen among individuals with less than secondary school. Moreover, according to this study, the odds of living with lifetime depression among individuals with any kind of post-secondary education is 1.54 times compared to individuals with lower than post-secondary education. Oquendo et al. (37) noted that being more educated and at the earlier age of onset of depression increased the risk for future depression in men. On the other hand, a study by Da Silva-Júnior et al. (26) is in opposition to these results—they noted that low educational level is one of the depression risk factors among men (OR = 3.03; *P* = 0.01). Also, dos Santos et al. (38) showed that the risk of depression decreases as the schooling years increase. This correlation is not unambiguous and requires further analysis. Individuals with higher educational achievement may be more prone to suicide risk when facing failures, public shame, and high premorbid functioning (39). Vijayakumar et al. (40) found an association between high education levels and high male suicide rates pointing to the fact that those with relatively high socioeconomic standing had the highest suicide rates.

A strong correlation is noticed between alcohol abuse and depressive disorders in men (2). They often use alcohol to cope with their mental pain. Our study confirms this dependency—one of the risk factors related to the level of depression among men with diagnosed depression is alcohol abuse/addiction. These factors do not occur in the control groups, so it is specific for men with depression. A study by Bazargan-Hejazi et al. (41) showed that alcohol overuse is one of the predictive characteristics of depression in men. Moreover, men who overused alcohol were 2.5 times more likely to report greater depression (95% CI = 1.37–4.45). Data from the National Comorbidity Survey estimated the lifetime prevalence of major depression to be nearly one quarter (24.3%) among alcohol-dependent men exceeding the prevalence rates among individuals without alcohol use disorder. In clinical samples, the lifetime rates of co-occurrence are greater still, ranging from 50 to 70% (42).

According to Richards and Sanabria (43) and Hamano et al. (44), risk factors associated with depression included the history of family depression and alcohol overuse. A review and meta-analysis of the genetic epidemiology in major depression have indicated that major depression is considered a familial disorder, which mostly or entirely results from genetic influences (45). Moreover, the risk for major depressive disorder was highest among grandchildren with two previous generations affected by the disorder, suggesting the potential significance of a family history of depression beyond two generations (46). A study of Angelini et al. (47) noted that individuals who were exposed during childhood to a parent with mental health problems suffered from depressive symptoms more often in late adulthood than those who were not (OR, 1.76; 95% CI, 1.43–2.17). Our study is noting that the occurrence of mental disorders in the family is one of the risk factors related to the level of depression in healthy men and men with depressive disorders. Suicidal behavior remains a risk factor in men with diagnosed depression. Family history of suicidal acts tripled the risk of future suicidal acts. Suicidal behaviors cluster in families, independently of the transmission of psychiatric conditions (34). A large epidemiologic study (48) showed that a family history of psychiatric disorders increased the risk of suicide completion. In a meta-analysis of Carrasco-Barrios et al. (49), significant OR for family history of mental disorder when considering all types of suicidality and suicidal ideation was obtained. A systematic review of Hawton et al. (10) noted that male gender (OR, 1.76; 95% CI, 1.08–2.86) and family history of psychiatric disorder (OR, 1.41; 95% CI, 1.00–1.97) are factors significantly associated with suicide.

Parenthood is associated with physical and mental health. Wang et al. (50) found that the number of children was not associated with a decreased prevalence of depression in men. Some studies showed that under certain conditions (i.e., unemployment), married fathers living with minor children report more distress than mothers (51). The study of fathers at age 18–40 revealed major depression symptoms more frequently in those men that became a father during adolescence (52). In our study, having children turned to be a risk factor related to the level of depression among depressive men. We found a correlation between the number of children and the risk of depression in patients from the control group of healthy men. Results of Mirowsky and Ross (53) showed that the later in life men become fathers, the more they seem to benefit emotionally from being a parent—they are less depressed the longer they delayed parenthood.

Mood disorders like recurrent depressive disorder or depressive episode may be an important factor contributing to the negative assessment of ability to cope with difficult situations and a greater tendency to perceive stressful events as overwhelming. Patients with depression more often use ineffective and avoidance strategies to cope with stress compared to healthy controls (54). A study by Walker et al. (55) showed that depressive symptoms were directly related to the use of less adaptive coping methods and directive instrumental social support. According to Nadler et al. (56), men sought help more often for instrumental-informational reasons. Shell and Eisenberg (57) demonstrated that instrumental (or direct) support did not result in feelings of threat, low perceived control, or high dependency in boys. In our study, seeking instrumental support in coping with stress remains depression and suicidal behavior risk factor characteristic for men with diagnosed depression. Kelly et al. (58) and Matud et al. (59) also noted that men use more problem-focused or instrumental methods of handling stressful experiences. Those results differ from other studies on this topic published recently. In the study of Liang et al. (60), instrumental support [Adj' OR = 0.90, 95% CI (0.84– 0.97)] was significantly less likely to be used by the group of patient with attempted suicide, but it has to be kept in mind that about 68% of the studied group consisted of women. Moreover, according to Ambrus et al. (61), only avoidant coping may be associated with increased suicide risk in psychiatric patients independently of a history of attempted suicide. The topic of male coping strategies should be investigated in the future.

A group of depression and suicidal behavior risk factors, including student status, low satisfaction with the financial situation, having children/more than one children, the occurrence of mental disorders in the family, unemployment, alcohol abuse, and seeking instrumental support should be analyzed by the therapists, doctors, and other professional staff engaged in the therapy of men who can suffer from depression.

Our study shows that active coping strategy and living in a middle-size city are two common protective factors against depression for men. There are some positive factors related to the level of depression that are characteristic only for men with depressive disorder: vocational education, the use of religious practices to deal with stress, and presenting social competencies. Some of these factors coincide with protective factors against suicidal behavior, which are common for all men: active coping, living in a middle-size city, and personal and social competencies. We also selected a group of protective factors against suicidal behavior characteristics for men with depression: bachelor status and using religion to cope with stress.

There are numerous studies on protective factors against depression and suicide. A study by Breton et al. (62) showed that productive coping is one of the protective factors against depression and suicidal behaviors. Our results confirm this association in males—active coping with stress is a very important protective factor against both depression and suicidal behavior. It should be noted that active coping with stress (engagement coping) involves active dealing with stressors or related emotions, whereas disengagement coping involves escaping stressors or subsequent emotions. Engagement coping is associated with improved well-being, and disengagement coping is associated with negative mental health outcomes (63). Active coping with stress is a factor of protection against a negative mental state, and thus against depression.

The living environment seems to have neurobiological effects that contribute to different course and outcome of psychiatric disorders (64). Research has also found that urbanity/rurality shapes intra-regional differences in suicide (65). According to a recent study by Barry et al. (66), men living in rural areas are more likely to effectively commit suicide. There are many possible explanations for increased suicide risk in rural areas. Despite popular clichés about anonymous city-dwelling, rural living can lead to social isolation, resulting in less intimate face-to-face contact with family and friends, which, in turn, increases the risk for suicidal behavior (64). Rural dwellers have easier access to lethal means, which increases their suicide risk (67). Country living is often related to a lower socioeconomic status as well as stigmatized attitudes toward visiting mental healthcare facilities [e.g., general practitioner (GP) and psychiatrists], and long travel distances diminish the accessibility to specialized healthcare providers (68). Several empirical studies emphasized an elevated vulnerability in rural areas, whereas others drew an opposite conclusion (66). In our study, living in middle-size and small cities is significantly more protective against depression among men than living in large cities. According to male suicidal behavior, living in middle-size cities has a protective dimension. Poor social networks such as residence in a rural location and having few contacts with friends were the identified risk factors of depression in late life (69). Another study emphasized that rural areas upheld traditional gender roles. Consequently, men who cannot fulfill their roles may experience increased stress. Men showed susceptibility to a wider range of stressors (e.g., financial stress) as compared with women (70).

Results of Omary research (71) suggested that higher education levels might play a protective role against suicidal ideation among men with depression. Our results show an opposite correlation—vocational education is a statistically significant positive factor related with the level of depression in men with diagnosed depression. What is interesting, the same factor turned to be a depression risk factor in a group of healthy men. To explain this discrepancy, one should thoroughly analyze the degree of satisfaction with a patient's work. The research results show that men are more sensitive than women to work stress, work problems, and financial problems. These factors may be more important than the level of education itself. Additionally, there is still a belief among the society that a man should be responsible for the maintenance of his family. This stereotype may increase stress related to the performed work and negatively affect the male mental state, including increasing the risk of depression (70). Therefore, a simple conclusion of the results of the correlation between education and depression should be taken with caution.

An extensive review of Koenig et al. (72) showed that religion and active participation in religious practices have the power to neutralize life stress and might help both to prevent the onset of depression, and if depression develops, they shorten the time it takes to resolve. A recent study by Arjan et al. (73) showed that religiosity tended to be more protective in persons with psychiatric symptoms than in people with physical illnesses. A study of McFarland (74) presented that organizational religiosity decreases symptoms of depression and increases levels of optimism and self-esteem over time for men. Religion provides men a unique context in which they were able to reap more benefits from religious activities. Organizational religious activity (i.e., involvement in religious services, bible groups, and prayer groups) appears to have a more pronounced impact on mental health for men. Religious attendance was protective for men (75). Our study shows that applying religion to cope with stress has a protective dimension in a group of men with depression disorders. These results stay in contradiction to the findings of Orr et al. (76)—they found that higher initial religious engagement is associated with higher initial depressive symptoms, especially among men. A systematic review that examined the correlation of religion and suicide conducted by Koenig et al. (77) found that most of the studies (84%) indicated fewer suicides or more negative attitudes toward suicide among the more religious people. Recent research suggests that religion prevents suicide primarily through religious doctrines that prohibit suicide (78). A systematic review of Lawrence et al. (79) seeking to identify the specific dimensions of religion (participation, affiliation, doctrine) that are associated with specific aspects of suicide (ideation, attempt, completion) found that religious affiliation does not necessarily protect from suicidal ideation but does protect from suicide attempts. It further found that religious service attendance is not protective against suicidal ideation but does protect from suicide attempts and may also have a protective effect against effective suicide. The protective effect of religion was noted by Kralovec et al. (80) especially against the capability aspect of suicide among men. Further research is warranted in the area of religion, depression, and suicidality to better understand the relationship between religiosity and gender. Also, the influence of religion on the various groups within the dominant religions remains under-investigated (81).

Results of our study indicate that social competencies are positive factors related to the level of depression and suicidal behavior among depressive males, while personal competencies have a protective dimension of male suicidal behavior. It is believed that the post-aggregation of one's social competencies may depend on the mood of a depressed person. It has been noted that people with depression tend to negatively assess their own competencies in comparison to people without depression. Moreover, people with depression also rate their partners as less competent than people without depression (82). Other researchers also emphasize the negative style of social functioning of patients with depression—these patients have impaired social communication (impaired emotion recognition, diminished cooperativeness) and impaired social perception (reduced empathy, theory-of-mind deficits) and their impact on social networks (83). Bearing these results in mind, we can explain the obtained results of our research. Similar observations were noted as personal competencies are concerned, which are directly related to social competencies because they affect the quantity and quality of relationships created with others. They enable engaging in effective social interactions and are associated with resilience and positive adaptation to the environment in which the individual lives. Therefore, it is a protective factor against the development of depression and suicidal thoughts (84).

Most of the scientific reports emphasize that marriage appeared protective against suicide especially in men (85). Suicide data by the year 2019 published by Polish National Police show that about 37% of men who committed suicide were bachelor and 43% were married. The lowest number of suicides was among widowers (8%) and divorced/separated men (11%) (4). Similar statistics presented by Park et al. (86) show that the highest relative suicide risks occur in never-married men aged 55–64 years. Also, the study by Augustine et al. (87) noted that couples in separation were exposed to suicide risk that was nearly 52% greater than that of the married. In the study of Griffiths et al. (88) for single and divorced men aged 25 and over, suicide rates were around three times higher than for married men throughout 1983–2004. Results of Omary (71) suggested that marriage might play a protective role for suicidal ideation among men with depression. Our results present a completely different dimension—bachelor status is a characteristic protective factor for suicidal behavior among men with diagnosed depression. Discrepancies that were noted should be interpreted with caution as they do not take into account the full characteristics of the study subject. e.g., social situation and social competencies are not known. Our study also emphasizes the need for a broader look at the risk factors of depression in men, including protective factors. This is because they are not isolated factors, but often interact.

Factors positively related to the intensity of depression should be checked in future studies to see if they constitute a factor in the development of depressive disorders throughout life. The role of factors, the negative relationship of which with the level of depression has been shown in the study as potential protective factors, also needs to be checked.

## Limitations

Our study is not without limits. First of all, researchers used self-report methods, so questionnaires were self-filled. However, study participants completed questionnaires at the hospital. The second important limitation of the study is voluntary participation in the study. This means that only some of the patients agreed to participate in the study. This could have influenced the results obtained. Another limitation is that there is no information about the co-occurring somatic and psychiatric disorders among the groups and also specific somatic disorders among CG1. Men from CG2 self-evaluate their physical and mental health. It could also have influenced the obtained results.

## Conclusion

The risk factors identified in this study should be included in the clinical assessment of depression and suicidal behavior risk among male patients. Further large-scale studies are required to clarify the role of some factors. They may improve prognostic accuracy in major depression and suicidal behavior among men. There are some protective factors, including productive coping and personal and social competencies, which can be developed and should be especially considered and strengthened in mental health promotion programmes aimed at men.

## Data Availability Statement

The original contributions presented in the study are included in the article/supplementary material, further inquiries can be directed to the corresponding author/s.

## Ethics Statement

The studies involving human participants were reviewed and approved by Bioethics Committee of Warsaw Medical University No. KE-0254/335/2015. Written informed consent for participation was not required for this study in accordance with the national legislation and the institutional requirements. Written informed consent was not obtained from the individual(s) for the publication of any potentially identifiable images or data included in this article.

## Author Contributions

All authors listed have made a substantial, direct and intellectual contribution to the work, and approved it for publication.

## Conflict of Interest

The authors declare that the research was conducted in the absence of any commercial or financial relationships that could be construed as a potential conflict of interest. The reviewer AK declared a shared affiliation with several of the authors, AK, MJ, AM, and BW-Z, to the handling editor at time of review.
